# The Guatemala-Penn Partners: An Innovative Inter-Institutional Model for Scientific Capacity-Building, Healthcare Education, and Public Health

**DOI:** 10.3389/fpubh.2017.00070

**Published:** 2017-04-10

**Authors:** Maria Alejandra Paniagua-Avila, Elizabeth Messenger, Caroline A. Nelson, Erwin Calgua, Frances K. Barg, Kent W. Bream, Charlene Compher, Anthony J. Dean, Sergio Martinez-Siekavizza, Victor Puac-Polanco, Therese S. Richmond, Rudolf R. Roth, Charles C. Branas

**Affiliations:** ^1^Perelman School of Medicine, University of Pennsylvania, Philadelphia, PA, USA; ^2^School of Medicine, Universidad Francisco Marroquín, Guatemala City, Guatemala; ^3^Department of Dermatology, University of Pennsylvania, Philadelphia, PA, USA; ^4^School of Medicine, Universidad San Carlos de Guatemala, Guatemala City, Guatemala; ^5^Department of Biostatistics and Epidemiology, Perelman School of Medicine, University of Pennsylvania, Philadelphia, PA, USA; ^6^Department of Family Medicine and Community Health, Perelman School of Medicine, University of Pennsylvania, Philadelphia, PA, USA; ^7^Office of Global Health and the Provost’s Office of the University of Pennsylvania, Philadelphia, PA, USA; ^8^Department of Biobehavioral Health Sciences, University of Pennsylvania, School of Nursing, Philadelphia, PA, USA; ^9^Department of Emergency Medicine, Perelman School of Medicine, University of Pennsylvania, Philadelphia, PA, USA; ^10^Department of Orthopedic Surgery, Universidad Francisco Marroquín, Guatemala City, Guatemala; ^11^Department of Epidemiology, Columbia University Mailman School of Public Health, New York, NY, USA

**Keywords:** Guatemala, global health, capacity building, scientific diplomacy, partnership

## Abstract

Population health outcomes are directly related to robust public health programs, access to basic health services, and a well-trained health-care workforce. Effective health services need to systematically identify solutions, scientifically test these solutions, and share generated knowledge. The World Health Organization (WHO)’s Global Healthcare Workforce Alliance states that the capacity to perform research is an essential factor for well-functioning public health systems. Low- and middle-income countries have greater health-care worker shortages and lower research capacity than higher-income countries. International global health partnerships between higher-income countries and low-middle-income countries aim to directly address such inequalities through capacity building, a process by which human and institutional resources are strengthened and developed, allowing them to perform high-level functions, solve complex problems, and achieve important objectives. The Guatemala–Penn Partners (GPP) is a collaboration among academic centers in Guatemala and the University of Pennsylvania (Penn), in Philadelphia, Pennsylvania that echoes the vision of the WHO’s Global Healthcare Workforce Alliance. This article describes the historical development and present organization of the GPP according to its three guiding principles: university-to-university connections, dual autonomies with locally led capacity building, and mutually beneficial exchanges. It describes the GPP activities within the domains of science, health-care education, and public health, emphasizing implementation factors, such as sustainability and scalability, in relation to the guiding principles. Successes and limitations of this innovative model are also analyzed in the hope that the lessons learned may be applied to similar partnerships across the globe.

## Introduction

Health is improving more slowly in many low- and middle-income countries (LMIC) than in nations with more resources, increasing health disparities around the globe (1). Population health outcomes are directly related to robust public health programs, access to basic health services, and a well-trained health-care workforce (2). Therefore, the World Health Organization (WHO) recommends that a country maintain no less than 2.28 workers per 1,000 population in order to achieve basic health-care coverage (3). Based on this statistic, there is a global shortage of 2.4 million doctors, nurses, and midwives (3). As the burden due to non-communicable diseases grows in LMIC, this shortage will become a major limitation for expanding the scope of health systems to address health needs (2).

The improvement of public health systems goes beyond increasing the number of health workers. Effective health services need to systematically identify, develop and test solutions, and finally share and apply the generated knowledge (4). Therefore, the WHO states that the capacity to perform health research is an essential factor of public health systems (1). Unfortunately, LMIC have extremely low expenditures in research compared to higher-income countries and the density of researchers per population is 1,000 times lower than in developed nations (4). Global health partnerships between higher-income countries and LMIC aim to directly address such inequalities by building capacity, a process by which human and institutional resources are strengthened and developed, allowing them to “perform functions, solve problems and achieve objectives” (5).

The Guatemala–Penn Partners (GPP) is a collaboration among academic centers in Guatemala and the University of Pennsylvania (Penn), in Philadelphia, Pennsylvania that echoes the vision of the WHO’s Global Healthcare Workforce Alliance (6). This article describes the historical development and present organization of the GPP according to its three guiding principles: university-to-university connections, dual autonomies with locally led capacity building, and mutually beneficial exchanges. It discusses the implementation of activities in the domains of science, health-care education, and public health in relation to such guiding principles. We analyze the successes and limitations of this innovative model with the hope that the lessons learned may provide a useful model for similar partnerships across the globe.

## Background and Rationale

Guatemala is a multilingual and multicultural Central American country with 22 ethnic groups that speak 23 separate indigenous languages in addition to Spanish. Forty percent of its population is ethnically indigenous Maya and the rest are non-indigenous or of mixed indigenous and European ancestry (7). Although Guatemala is a LMIC, most of Guatemala’s indigenous, rural populations live on less than one United States dollar per day (8, 9). Health disparities, poor education, and racial discrimination disproportionately affect the indigenous and rural populations (10, 11). Guatemala’s first democratic elections were held in 1985 during a civil war that lasted 36 years. Although the Peace Accords were signed in 1996, the country continues to be affected by political instability, corruption, and slow moving institutions (11, 12). The rates of interpersonal violence still remain one of the highest in the world outside of active theaters of war (12).

Guatemala is currently undergoing an epidemiologic transition in which it continues to struggle with communicable, maternal, neonatal, and nutritional issues while facing new epidemics of non-communicable illnesses and injuries that are some of the highest in the world (13, 14). Adding further challenge to the poor health predictors, there are only 1.25 health workers per 1,000 population in Guatemala, a number that is significantly lower in the rural areas, the lowest in Central America, and approximately half of the WHO recommendations (15).

Guatemala’s training institutions for health-care and scientific education consist of one public university and seven private universities. In 2005, the Interinstitutional Commission of the Academic and Health Sectors was formed with the goal of improving Guatemalan public health by leveraging public and academic resources (16). However, the national expenditure in research and development is one of the lowest ones in Central America and the number of researchers and scientific publications is significantly lower than many other Latin American countries (17).

The Penn is located in Philadelphia, United States of America (USA), a city affected by socioeconomic and health disparity challenges akin to those of Guatemala. Philadelphia has some of the worst health indicators, poverty, and violence rates of the largest USA cities. Over 40% of its population is ethnically Black non-Hispanic and the remainder is Asian, Hispanic, and White (18). Black populations in Philadelphia are medically underserved and disproportionately affected by socioeconomic and health challenges such as premature death, smoking, diabetes, and violence (18).

## Description of the Case

Historical ties between Guatemala and Penn have existed for almost a century. Beginning in 1930, scholars from Penn traveled to Guatemala to study its culture, linguistics, and ancient artifacts. In 1970, the Penn Museum developed the “Pennsylvania Declaration” which worked to end the practice of removing native archeological objects from their countries of origin, including Guatemala (19). The declaration established as an ideal the responsibility of the researcher to respect the autonomy of the studied country. Although imperfect in practice, the declaration served an important function in laying a foundation of integrity upon which the collaboration of the GPP program rests.

Building upon this important doctrine, a group of orthopedic surgeons from Penn and one of the private Guatemalan universities, Universidad Francisco Marroquín (UFM), created an exchange program for medical residents during the 1980s. Its purpose was to promote experiential and knowledge interchange among Penn and Guatemalan medical residents, with a focus on violence and injury, as Guatemala was recovering from a civil war. Participants used to visit and practice medicine for a short period of time at one of the Guatemalan public hospitals or at one of the Penn health centers. A group of Penn academicians and Guatemalan individuals recognized the importance of added scientific collaborations to such exchanges and endowed the partnership with a renewed emphasis on scientific and health capacity building.

As a result, in 2005, the GPP was founded, with the participation of UFM, Universidad de San Carlos de Guatemala (USAC), the public Guatemalan University, and Penn. To formalize the partnership, Memoranda of Understanding were signed among the participant institutions. In addition, two faculty members, one from UFM and one from USAC, were contacted by Penn to represent and run the GPP in Guatemala. Continuing the focus on violence and injury, the first of three National Institutes of Health (NIH) Fogarty International Center (FIC) Training grants were obtained, which facilitated the training of the first cohort of Guatemalan scientists. As the GPP gained support and credibility, additional individuals representing multiple disciplines and university departments in Guatemala and at Penn became active participants, creating new interventions, and collaborating with existing ones. Ten years later, two other private Guatemalan universities, the Universidad del Valle de Guatemala and Universidad Rafael Landívar entered the GPP. In addition, the GPP maintains a connection with the Guatemalan Ministry of Health (MOH) through USAC and collaborates with multiple municipalities, private and public hospitals, the Instituto Guatemalteco de Seguridad Social Institute of Guatemalan Social Security health system, and non-governmental organizations (NGOs).

The GPP today is a multidisciplinary platform that facilitates communication among professionals in public health, anthropology, business, dentistry, engineering, medicine, nursing, nutrition, and other domains of the arts and sciences. The GPP echoes the vision of the WHO’s Global Health Workforce Alliance strategies of partnerships and health-care education (2, 6).

## Methodological Aspects

**Figure 1:**
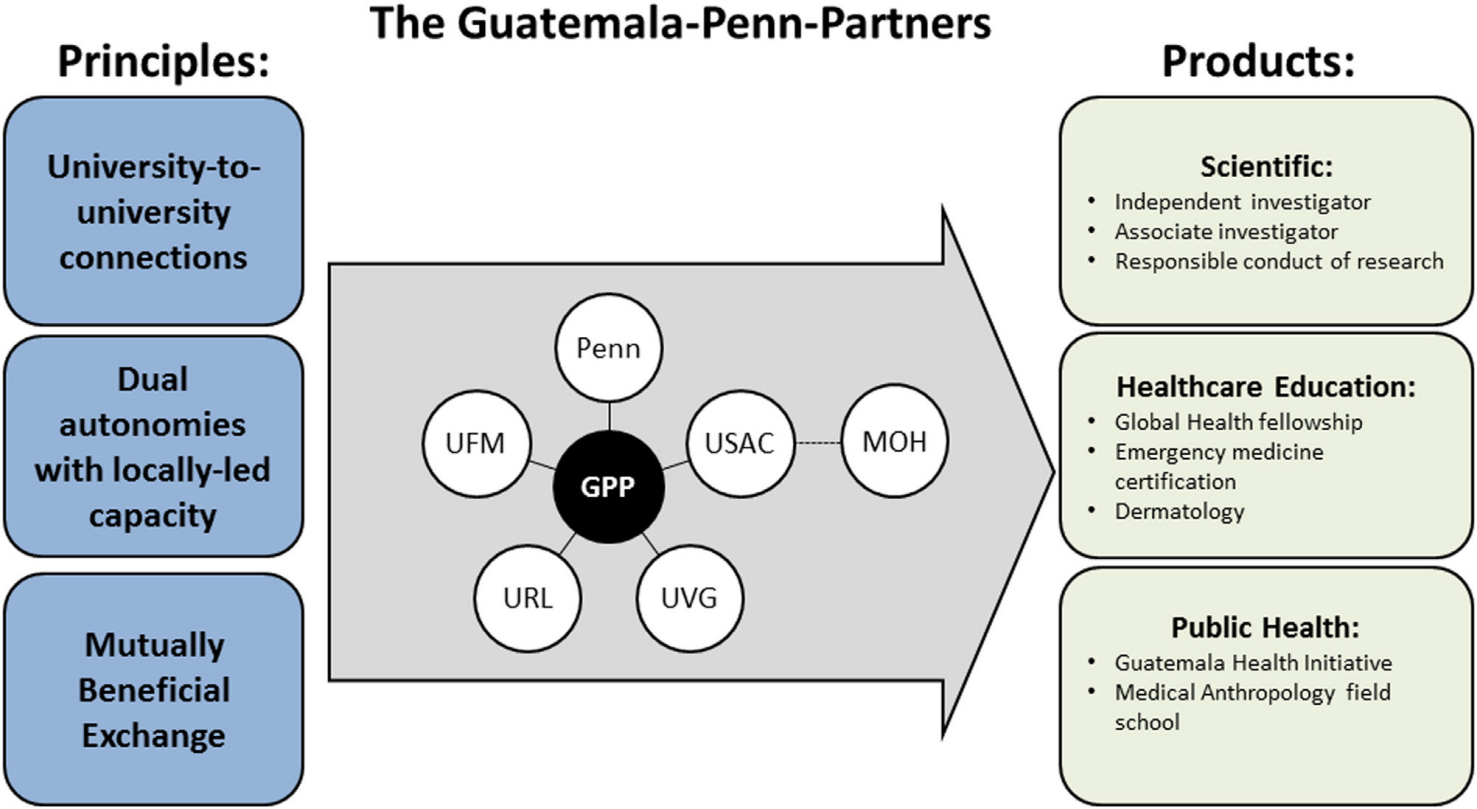
**GPP principles, university members, and products**. The GPP principles guide the collaborations between the main university members and the development of products of scientific, health-care education, and public health nature. GPP, Guatemala–Penn partners; Penn, University of Pennsylvania; UFM, Universidad Francisco Marroquín, Guatemala; USAC, Universidad de San Carlos, Guatemala; URL, Universidad Rafael Landívar, Guatemala; UVG, Universidad del Valle de Guatemala; MOH, Ministry of Health, Guatemala.

The GPP is founded on the principles of university-to-university connections, dual autonomies with locally led capacity, and mutually beneficial exchanges. The partnership promotes collaborations within the domains of scientific capacity building, health-care education, and public health. Figure 1 provides an overview of the principles, main partners, and initiatives of the GPP. Table 1 shows the starting year, priorities, and main entity targeted by the GPP initiatives.

**Table 1:** **GPP initiatives**.

Domains	Initiatives	Start	Priority	Main entity targeted
Scientific	Independent investigator	2006	Research and public health leadership development in Guatemala	Individuals and universities
	Associate investigator	2006	Research user training in Guatemala	Individuals and universities
	RCR	2014	RCR development in Guatemala	Individuals and universities
Health-care education	Global health fellowship	2013	Global health training for University of Pennsylvania (Penn) PCP	Individuals
	Emergency medicine (EM) certification	2017	Institutionalization of EM residency in Guatemala	Universities and Public Health Hospitals
	Dermatology	2010	Dermatology training and cultural exchange for Penn and Guatemalan residents	Individuals and health-care facilities
Public health	Guatemala Health Initiative	2005	Development of community-based health interventions in rural Guatemala	Individuals, health-care facilities, and communities
	Medical Anthropology Field School	2005	Multidisciplinary research training for Penn students	Individuals

*The GPP initiatives, classified within the scientific, health-care education, and public health domains, address health priorities in Guatemala or at Penn and target unique entities*.

*RCR, responsible conduct of research; PCP, primary care providers*.

### Central Principles of the Guatemala–Penn Partners Program

#### University-to-University Connections

The GPP is principally based on university-to-university connections rather than on interactions with non-academic, government, and NGOs. This is a key principle because universities tend to have historically established and ongoing stability that is often lacking in other types of institutions. Universities tend to be more resistant than other national institutions to political changes or fluctuations in funding. As such, universities are often enduring partners and university-to-university connections may lead to more sustained, long-term relationships. Additionally, universities facilitate the participation of individuals and departments from multiple disciplines, allowing for the creation of initiatives that follow a diverse range of methodologies that complement and synergize each other.

#### Dual Autonomies with Locally Led Capacity

The GPP methodologies involve recognizing the local capacity and respecting the autonomies of the parties involved. Recognizing the local capacity involves the identification and appreciation of the local human and institutional resources. The GPP accomplishes these goals by operating under the principle of dual autonomies, which means that Guatemala’s and Penn’s respective resources remain under intrinsic control. Each party may therefore choose to share resources, emphasizing the continued autonomy of the local owner, and resources will never be taken without permission. In addition, the principle of dual autonomies means that each of the parties involved is independent and self-controlled. Through this method, the GPP echoes the Pennsylvania Declaration’s premise that the best and most sustainable scientific collaborations respect the local capacity by assuring the autonomies of all partners.

#### Mutually Beneficial Exchange

The principle of mutual exchange refers to the concept that all GPP participants benefit from the sharing of ideas, spaces, cultures, and resources to promote scientific and health-care capacity. This principle acts to ensure that benefits are accessible to “all its parties, at every level” (19). In recognition of the similarity of issues faced by Penn and Guatemala’s communities, the GPP therefore believes that each exchange has the potential to serve as a mutually beneficial learning opportunity for each party as they study and attempt to correct shared issues. While the GPP does acknowledge the disparity in resources either party has in order to face such struggles, this approach encourages constant bilateral learning and exemplifies the respect each partner holds for the other. There must be a mindset of readiness to contribute and learn from the challenges of the partner institutions and countries. It is important to note that the academic and educational resources that Guatemalan institutions may receive from their northern partner may be equaled or exceeded by the benefit for the USA institution of the opportunity to work in a low-middle-income country and build its reputation in the field of global health.

### Guatemala–Penn Partners’ Initiatives in the Domains of Scientific Capacity Building, Health-care Education, and Public Health

#### Scientific Initiatives

##### Independent Investigator Program

The mission of the independent investigator program is to prepare Guatemalan researchers and public health professionals for successful careers as principal investigators and program leaders. By focusing on chronic illnesses or injury and violence, this 2-year program targets the main causes of morbidity and mortality in Guatemala. Candidates consist of motivated physicians and other clinicians who are seeking to access higher education that is currently unavailable in Guatemala in order to advance their careers. During the first year of the program, students enroll and complete the course load of the Masters of Science in Clinical Epidemiology or the Masters of Public Health (MPH) departments at Penn. Simultaneously, participants must plan a research thesis relevant to health within Guatemala and are assigned a mentoring team. Students return to Guatemala during the second year of their training to implement the research project and embark on fieldwork. They receive a Penn diploma upon completion of their thesis.

The independent investigator program is designed to promote careers in research. By fostering such careers, it is hoped that research capacity will be built in a sustainable and self-perpetuating manner. Trainees are instructed in scientific and grant-writing instruction, presentation skills, and responsible conduct of research (RCR) training. Since its initiation in 2010, a dozen Guatemalans have been trained as independent investigators, producing almost 20 peer-reviewed publications. One GPP Fogarty trainee also received the Lancet Outstanding Global Health Research Project of the Year in 2011 for his cross-sectional time series study of violence and mental health during and after the civil war using data from the Guatemalan mental health survey (20).

##### Associate Investigators

The associate investigator certification seeks to train Guatemalan health researchers through a part-time program taught in Guatemala over 6 weeks. Participants include health workers, educators, and managers. Courses are taught in Spanish by USAC, UFM, and Penn faculty, as well as previous independent investigators. Participants learn about biostatistics, epidemiology, RCR, and appraisal of the scientific literature. Three dozen Guatemalans have participated in this program, including two university deans and a university president. Associate investigators may complement their professional careers with research skills or may choose to have collaborative roles in research.

##### Responsible Conduct of Research

The RCR program aims to develop a research ethics knowledge base in both Guatemalan Universities and Penn. It seeks to professionalize the capacity of institutional review boards (IRBs) in Guatemala and to enhance the sensitivities of the Penn IRB to the RCR in low-middle-income nations. The program trains USAC and UFM faculty, graduate students, and staff in RCR, teaching, and evaluation to build educational programs at the Guatemalan universities. It is also training a cohort of Guatemalan IRB administrators. Enrolled fellows receive basic RCR training in Guatemala, followed by a 6-month fellowship at the Human Research Protections Program at Penn. Upon their return to Guatemala, fellows participate in a field practicum at a local IRB. Dozens of Guatemalan have additionally been trained by this NIH FIC grant.

#### Health-care Initiatives

##### Global Health Fellowship in Comprehensive Health

The Department of Family Medicine and Community Health at Penn created the Global Health Fellowship in Comprehensive Health, in concert with the GPP, in 2013. This fellowship aims to develop clinical, educational, and leadership skills in the care of underserved patient populations in the western highlands of Guatemala and in Philadelphia. Primary care physicians from the USA can enroll in a 1- or 2-year program. Fellows enrolled in the 1-year track receive a global health certificate. The 2-year program provides an MPH degree with a concentration in global health. Both tracks require equal periods of clinical service in Guatemala and Philadelphia. This way, fellows experience the culture of both places and can elect to participate in local educational activities or research projects related to their interests. Fellows receive training at Penn through the MPH program, and serve as educators in the international educational exchange program in the Hospitalito Atitlán for Guatemalan physicians most of whom are not residency trained. To date, four fellows have been enrolled.

In addition, a continuing medical education course taught at Hospitalito Atitlán provides cultural immersion and medical Spanish for health-care providers from the USA and opportunities for cultural exchange and clinical lectures for the Guatemalans. Courses include medical terminology, Guatemala health-care systems, health-seeking behavior among Guatemalan Mayans, and impact of the history of violence and social disruption on health. Course teachers include a Mayan physician, language educators from Guatemala and USA physicians.

##### Emergency Medicine (EM) Certification

Penn’s Department of EM has been involved with the Guatemalan health-care initiatives since 2003 in the form of teaching missions and disaster relief efforts (21). Through continued interactions, it was recognized that emergency treatment in Guatemalan regional hospitals was often led by providers in their senior year of medical school or in their first year out of residency. In larger teaching hospitals, providers come from a wide array of medical fields and have limited EM career experience. By contrast, EM as a medical specialty has been established since the 1970s in the USA and providers receive postgraduate residency training in the management of a diverse range of undifferentiated emergency illness.

In order to increase emergency care in Guatemala, Penn faculty had ongoing interactions with leading physicians, mostly at USAC, about the need for EM certification in Guatemala. Guatemalan and Penn staff have discussed the history and current needs and practices of emergent care in their respective settings. Frameworks were discussed to make EM a formal department, and Penn faculty members were given Affiliate Professor appointments at USAC, according them with official status as teachers and eligibility for clinical practice in Guatemala. Since then, partnerships have been forged with other USA entities interested in global health and international EM, including Wayne State University in Michigan, the International Section of the American College of Emergency Physicians, and the International Federation of Emergency Medicine.

From among the group of physicians currently working in emergency services in Guatemala, leaders from surgery and pediatrics have volunteered to be the directors of the USAC EM residency program. In its special role as the only medical licensing agency in Guatemala, USAC has approached the MOH and the Social Security Institute for approval of the new specialty as well as for funding of EM residency training slots. In support of this effort, the USA government has awarded a nine-month Fulbright Scholarship to allow for an extended period of activity in country for the purpose of curriculum development, training the first group of residents, mentorship of faculty members, mechanisms for specialty certification, establishment of a specialty society, and arrangement for meetings. At the time of the writing of this article, the matriculation of the first class of EM residents is anticipated in 2017.

##### Dermatology

In 2010, faculty of Penn’s Department of Dermatology established a partnership between Penn and the Instituto de Dermatología y Cirugia de Piel (INDERMA), the sole dermatology residency program in Guatemala. Through this program, INDERMA residents and attending physicians rotate through Penn’s clinical settings. In addition, each year two Penn dermatology residents, and two attending physicians, join INDERMA staff and residents to establish a week of clinics in rural communities in the Western Highland of Guatemala. The first rural clinic delivered dermatologic care to over 300 patients in seven sites. In addition, Penn helped to develop a teledermatology system, through which Guatemalan physicians are able to use smart phones to exchange medical information from one site to another, as well as with physicians in the US and at Penn. The goals of this technology are to develop a network between INDERMA, other Guatemalan dermatologists, and Penn dermatologists to share difficult cases, assist in dermatology teaching in Guatemala, provide access to dermatology services in the rural clinics of Guatemala, to encourage further collaborative research, and to provide exposure to dermatologic pathologies uncommon in Philadelphia.

#### Public Health Initiatives

##### Guatemala Health Initiative (GHI)

The GHI was founded in 2005, becoming the local, community-based arm of the GPP. Health interventions are based on community health surveys and mixed-methods evaluations that are performed by a partnership between Penn and communities in the Western Highlands of Guatemala. The GHI works with local NGOs and municipalities to perform locally relevant public health projects and train Guatemalan and USA students in community health assessment and research skills. Specifically, Penn has partnered with the Hospitalito Atitlán, a local non-profit hospital that closed during the civil war and was decimated by repeated mudslides, but that has now been rebuilt and re-opened. Community assessments have revealed that diabetes and other non-communicable illness are reaching epidemic levels. In response, a team of local health-care workers and Penn faculty, supported by the World Diabetes Foundation, initiated a longitudinal program that aims to improve diabetes care through improved prevention, diagnosis, and treatment (22). Other community efforts address questions related to asthma, indoor air pollution, water and sanitation, skilled birth attendant training, and health-care strategic planning and networking. Through the GPP, numerous medical providers, undergraduate and graduate students from Penn, and some intermittent students from USAC and UFM have offered health services at Hospitalito Atitlán. The GHI has worked in three rural areas in Guatemala including: the Municipality of Santiago Atitlán at the Hospitalito Atitlán, the public health center in Comalapa, and the Clínica Bárbara in San Juan Sacatepéquez.

##### Medical Anthropology Field School

One of the primary activities of GHI has been an annual 10-week field school in medical anthropology, which mostly takes place in Santiago Atitlán, in the Western Highlands of Guatemala. These have included community health surveys every 6 years as well as projects investigating the baseline needs and impact of local initiatives. Core to the field school is the principle that the knowledge created belongs first to the local community and second to the broader scientific community as it is generalizable to medicine, community health, and global health that focuses on underserved populations. Field school participants work on projects and concerns identified through community needs assessments or through local municipal and public health leadership. These locally led projects recognize the dual autonomy of the community as well as the scientific autonomy of the learners. At the end of the field school, participants present their findings back to the community, documenting that they have learned skills, and benefiting the community in the area public health. Topic areas have included the ecology of motherhood and the role of traditional birth attendants in gaining skills in delivery, contamination with attention to food and waterborne illness, mental health and the impact of trauma on culture-bound and foreign-defined illness, indoor biomass combustion and the impact on wellness and respiratory health and asthma, nutritional practices throughout the lifespan, contraception, health economics, and care-seeking behaviors, the built environment, and the epidemiologic transition and the epidemic of diabetes.

## Discussion

The GPP is an example of a partnership between academic institutions from a LMIC and a higher-income country. Founded on a century-old relationship between Guatemala and Penn, the GPP was formalized in 2005 with a renewed emphasis on science and health. The GPP seeks to promote the capacity of health workers and researchers in Guatemala and at Penn by developing multiple synergistic initiatives within the domains of science, health-care education, and public health, following the WHO’s Global Health Workforce Alliance strategies. Since its inception, the initial three-partner program derived from an orthopedics exchange initiative and has evolved to now include five Guatemalan universities spanning many health sciences and clinical departments including medical anthropology, dermatology, family medicine, and EM. The GPP’s reach sustainability and scalability have been promoted by its three guiding principles.

The university-level connections have allowed for the development of a durable, yet flexible partnership, within a politically changing environment. Such academic foundation provides a steady source of leadership, a necessary factor to achieve the program’s sustainability. Academic centers gather individuals from multiple disciplines, a process that facilitates the creation of different types of initiatives that are needed to address the diverse health needs of Guatemala and Penn. This endows the partnership with the ability to upscale with ease and flexibility. As an example, the needs identified through previous initiatives are beginning to be addressed by recent activities, sometimes developed by new GPP participants. The independent investigator initiatives led by the epidemiology department at Penn highlighted the need of RCR in both countries, which triggered the creation of the RCR led by the bioethics department at Penn. Additionally, through continuous clinical interactions between Penn and the Guatemalan health facilities, the need for an EM certification was recognized, leading to the establishment of the first EM residency program in Guatemala. However, one weakness of this approach is the lack of immediate national involvement. The GPP circumvents this issue by maintaining a national connection with the Guatemalan health system through its connection with USAC and MOH.

Just as important as identifying the core partners of the collaboration, is how one goes about engaging with them. The principle of dual autonomies with locally led capacity dictates the methodology of the GPP. First, it establishes that all the GPP relationships are characterized by mutual respect and trust, which are essential elements of a successful global health partnership. Second, it capitalizes on locally derived leadership which helps to identify motivated individuals who then become essential partners of the GPP as they help implement the program initiatives on the ground. This principle promotes the sustainability and scalability of the GPP activities. As an example, the Medical Anthropology Field School emphasizes the importance of respecting the autonomy of the community’s human, intellectual, and financial resources. Penn students develop research projects that follow the Guatemalan community members’ suggestions. The obtained results are taken back to the community by holding meetings with local leaders, and the findings often guide initiatives that aim to address the identified health needs. Such initiatives take into account the available resources and seek to promote capacity of local human resources for health. Another example is the NIH-funded graduate programs, which have trained over 30 Guatemalan researchers, many of whom have returned to work at academic and public health centers in Guatemala, reaching an expanding network of Guatemalans. However, this program also highlights the challenges faced by the GPP Guatemalan partners. As independent investigators trained at Penn return to Guatemala, they are often restrained by an environment with limited financial resources, infrastructure, and human resources, a lack of scientific career structure and political instability. The fact that training individuals is only one of the essential steps toward improving health systems in LMIC is one of the greatest challenges faced by global health partnerships (4).

The principle of mutually beneficial exchanges is both a product and an example of the respectful GPP collaborations. An innate inequality of resources exists between Guatemala and Penn, as the Penn has relatively greater access to funding, educational facilities, and approachable mentorship. Similar disparities often lead to power differentials among global health partnerships that include a higher-income country and a LMIC. However, the GPP recognizes that Guatemala and Penn participants are able to advance their careers in different but mutually reinforcing ways. Cultural, academic, experiential, and interpersonal skills are important aspects to a global partnership, just as financial resources are. In general, Guatemalan participants benefit from the academic expertise and financial resources that are lacking in Guatemala. In turn, Penn participants conduct international research, gain global health skills, and experience a different culture, all of which are highly demanded skills in the USA academic centers (23). The scientific initiatives allow Guatemalans to access the research education provided by Penn, while Penn mentors gain teaching experience and disseminate their scientific work in international settings. The health-care education activities allow Penn and Guatemalan residents to obtain clinical knowledge, skills, and resources not available in their countries. Finally, the Public Health initiatives generate information about the health status of the Guatemalan communities and build health capacity at the community and institutional level while Penn participants obtain real-world community health and research skills. It should be noted, however, that similar partnerships may have the unintended consequence of pressuring LMIC partners to engage in compensatory exchanges. The GPP attempts to alleviate such feelings through the notion that both parties involved in an exchange may benefit despite being devoted predominantly toward improving the health-care of one partner. This serves to acknowledge the true audience of the GPP and other similar initiatives by creating equally valuable opportunities for exchanges, independent of either partner’s resources.

While the GPP remains imperfect, it continues to represent a sustainable and scalable model of a global health partnership that improves the scientific and health-care capacity among its participants. Key factors and recommendations are summarized in a list below.

## Lessons Learned/Recommendations

- Building a partnership founded on university-level connections allows for continuity within politically unstable environments and functional scalability as academic centers facilitate the incorporation of multidisciplinary groups of students and faculty. However, academic partnerships need not to exclude the local public health authorities in order to obtain their financial and administrative support and to avoid diluting their responsibility over national health issues.
- Recognizing the power differential between partners in LMIC and higher-income countries is a necessary initial step toward achieving a relationship that respects dual autonomies and promotes equitable collaborations.
- Basing a global partnership’s initiatives upon local leadership promotes local ownership, which increases sustainability.
- Investing in mutually beneficial initiatives, rather than in initiatives that primarily benefit one partner, increases the sustainability and scalability of the partnership by creating an incentive for individuals affiliated with both parties to participate.
- By identifying and training motivated and engaged individuals, the partnership creates an expanding network of trainers and trainees, which favors the sustainability, scalability and reach of the program.
- Training the right set of individuals is necessary, but not sufficient to improve health systems. The success of such individuals, and hence global health partnerships, also depends on systemic factors, such as a reinforcing environment with supportive local institutions and financial incentives, which are challenging and require long-term investments.

In conclusion, the GPP represents a collaboration between Guate-malan public and private universities and Penn that is founded on the principles of university-to-university connections, dual autonomies with locally led capacity building, and mutually beneficial exchange. Its ongoing initiatives in the domains of science, health-care education, and public health strive to fulfill the WHO’s Global Health Workforce Alliance strategies of partnerships and education. The goal of both describing and analyzing the success and limitations of these initiatives is to provide insight into strategies that can be adapted to other contexts in order to promote and strengthen similarly oriented global partnerships.

## Author Contributions

The following authors contributed substantially to the conception and design of work or acquisition, analysis, or interpretation of data for the work; provided final approval of the version to be published; provided agreement to be accountable for all aspects of the work in ensuring that questions related to the accuracy or integrity of any part of the work are appropriately investigated and resolved: MP-A, EM, CN, EC, FB, KB, CC, TR, SM-S, VP-P, AD, RR, and CB. In particular, design of the program: EC, FB, KB, SM-S, and CB. Authors responsible for research on the history of the program: MP-A, EM, CN, EC, FB, and CB. The following authors contributed to the draft and/or revisions of the draft for important intellectual content: MP-A, EM, CN, EC, FB, KB, CC, TR, SM-S, AD, RR, and CB. Particular contributions and revisions were made by the following: RR and CN to dermatology clinical initiatives; AD to emergency medicine clinical initiatives; CC and TR to the background information on Guatemala nutrition and community health initiatives; MP-A, EM, KB, CB, and FB on the field school of anthropology, clinical sites, and historical background of the partnership; EC, MP-A, KB, and SM-S on the Guatemalan health background data; EM, CN, and AP on the Philadelphia health background data. MP-A and EC designed Figure 1. AP designed Table 1. MP-A, EM, CN, EC, RR, and CB oversaw all drafts and revisions to the entire paper content in addition to the aforementioned roles.

## Conflict of Interest Statement

The authors declare that the research was conducted in the absence of any commercial or financial relationships that could be construed as a potential conflict of interest.

## Abbreviations

CME, continuing medical education; EM, emergency medicine; FIC, Fogarty International Center; GPP, Guatemala–Penn Partners; GHI, Guatemala Health Initiative; IGSS, Institute of Guatemalan Social Security, Instituto Guatemalteco de Seguridad Social; INDERMA, Instituto de Dermatología y Cirugia de Piel; IRB, institutional review board; LMIC, low- and middle-income countries; MPH, Masters of Public Health; MSCE, Masters of Science in Clinical Epidemiology; MOH, Ministry of Health; NIH, National Institutes of Health; NCD, non-communicable disease; NGO, non-governmental organization; RCR, responsible conduct of research; USD, United States dollar; USA, United States of America; UFM, Universidad Francisco Marroquín; USAC, Universidad San Carlos; UVG, Universidad del Valle de Guatemala; URL, Universidad Rafael Landívar; Penn, University of Pennsylvania; WHO, World Health Organization.
